# The Impact of Minimally Invasive Surgery in the Treatment of Ovarian Cancer in Young Patients

**DOI:** 10.3390/cancers17132098

**Published:** 2025-06-23

**Authors:** Marta Heras, Mikel Gorostidi, Octavio Arencibia, Lucas Minig, Lola Marti, Myriam Gracia, Arantxa Lekuona, Isabel Niguez, Blanca Gil-Ibañez, Berta Diaz-Feijoo, Ana Gomez, Ana Lara, Rosa Alvarez, Virginia Corraliza, Jose Carlos Vilches, Maria de Marino, Pluvio Coronado, Silvia Duch, Emilio Bayon, Iria Aparicio, Raquel del Moral, Gemma Mancebo, Iñaki Lete, Violeta Tetilla, Ignacio Zapardiel

**Affiliations:** 1 Hospital Universitario Santa Cristina, 28009 Madrid, Spain; mherasgarcia@gmail.com (M.H.); rm.alvarez.lopez@gmail.com (R.A.); 2 Hospital Universitario de Donostia, 20014 San Sebastian, Spain; 3 Biogipuzkoa Health Research Institute, 20014 Gipuzkoa, Spain; 4 Faculty of Medicine, Basque Country University, 48940 Leioa, Spain; 5 Hospital Universitario Materno Infantil, 35016 Canarias, Spain; 6 IMED Hospitales, 46100 Valencia, Spain; 7 Hospital Universitario Bellvitge, 08907 Barcelona, Spain; 8 Hospital Universitario La Paz, 28046 Madrid, Spain; dra_gracia@hotmail.com (M.G.); ignaciozapardiel@hotmail.com (I.Z.); 9 Hospital Virgen de la Arrixaca, 30120 Murcia, Spain; 10 Hospital Universitario 12 de Octubre, 28041 Madrid, Spain; 11 Hospital Clinic de Barcelona, 08036 Barcelona, Spain; 12 Hospital General de Segovia, 40002 Segovia, Spain; 13 Hospital Virgen de las Nieves, 18014 Granada, Spain; 14 Hospital Universitario Ramón y Cajal, 28034 Madrid, Spain; 15 Hospital Quirón Málaga, 29004 Málaga, Spain; jcvilches@sego.es; 16 Hospital Universitario de Salamanca, 37007 Salamanca, Spain; 17 Hospital Universitario Clínico San Carlos, 28040 Madrid, Spain; plujcoro@ucm.es; 18 Hospital Universitario Infanta Sofía, 28702 Madrid, Spain; 19 Hospital Clínico de Valladolid, 47003 Valladolid, Spain; 20 Complejo Hospitalario Universitario de Pontevedra, 36005 Pontevedra, Spain; 21 Hospital de Poniente, 04700 Almería, Spain; 22 Hospital del Mar, 08003 Barcelona, Spain; 23 Hospital Universitario Araba, 01009 Vitoria, Spain

**Keywords:** ovarian, cancer, surgery, laparotomy, minimally invasive surgery

## Abstract

Background: Surgery is the main pillar for the treatment of ovarian cancer patients. The improvement of minimally invasive surgery (MIS) has led to its application to a greater number of processes. However, MIS for ovarian cancer is still under debate, since the behavior of ovarian cancer and its pattern of spread make it difficult to assure a proper surgery with this approach. Our aim was to evaluate the advantages of MIS in the management of ovarian cancer among young patients. Methods: A retrospective study was conducted with the participation of 55 Spanish hospitals. We included all patients aged between 18 and 45 years old who were diagnosed with invasive ovarian cancer from 2010 to 2019. Histological diagnosis was mandatory in all cases. All patients underwent surgery, which could be the initial treatment or after neoadjuvant chemotherapy. Depending on preoperative workup and surgical expertise, the surgical approach was either laparotomic or MIS. All data regarding the perioperative details, as well as prognostic factors, were compared depending on the type of surgical approach. Results: A total of 1144 patients were collected, among them 867 (75.8%) underwent laparotomy and 277 (24.2%) MIS. Epithelial tumors were diagnosed in 992 (86.7%) patients and non-epithelial cancers in 152 (13.3%). Complications such as blood loss, length of surgery, and hospital stay were higher in the laparotomy group, and were statistically significant (*p* < 0.0001). Recurrences were present in 353 (30.85%) patients: 302 (26.4%) in the open surgery group and 51 (4.45%) in the MIS (*p* < 0.0001). Deaths were reported in 199 (17.4%) patients: 169 (14.8%) after open surgery and 30 (2.6%) after MIS (*p* = 0.0026). Conclusions: Minimally invasive surgery does not seem to negatively impact the prognosis of young patients with ovarian cancer. Its use has been mainly for staging surgery in early-stage tumors, proving its safety with statistically significant differences. Nonetheless, these results are highly influenced by the predominance of early-stage and low-burden disease tumors in the MIS group. Moreover, there are confounding variables that seem to influence these results, such as age, tumor size, FIGO stage, and histology, which are important to consider in order to choose the best surgical approach. When the selection of patients is adequate, MIS does not entail a worse prognosis than laparotomy for ovarian cancer surgery.

## Simple Summary

The laparotomic approach has been the standard of care for ovarian cancer surgery. Currently, the increasingly widespread of minimally invasive surgery has led to debate about its applicability for oncological purpose and, therefore, ovarian cancer treatment. The extent of the disease in ovarian cancer entails a peculiarity in the surgical radicality, which is mostly achieved by laparotomy. Studies have been conducted in order to establish indications for minimally invasive surgery for ovarian cancer. Results have proven its safety in selected cases, mostly in early- stage tumors and for staging purpose. More studies are needed to add evidence to the literature to confirm its safety and indications.

## 1. Introduction

Ovarian cancer is more frequently diagnosed in post-menopausal patients, and it is considered an aggressive tumor that is usually diagnosed at an advanced stage. Treatment for ovarian cancer is multimodal, and consists of a combination of surgery and adjuvant chemotherapy in the majority of cases. Surgery is the main pillar of treatment, and it is considered as the initial tool when the extension of disease makes its complete resection amenable [1]. After initial surgery, a great number of patients require postoperative chemotherapy, depending on FIGO stage and histology. In some cases, the extension of the disease or the baseline condition of the patients, mostly elderly patients, does not allow a complete surgery, so the treatment starts with neoadjuvant chemotherapy [2]. After three or four cycles, reevaluation is made in order to propose interval debulking surgery if complete excision is amenable or, on the contrary, to complete chemotherapy [3,4]. Despite all therapeutical efforts, ovarian cancer is still the first cause of death due to gynecological malignancies [5].

Classically, surgery for ovarian cancer was made by an extended laparotomy, which makes possible a proper examination and access to the upper abdomen [6,7]. The introduction of MIS has led to doubts regarding its applicability to gynecological oncology procedures [8]. The nature of ovarian cancer and its extension into the abdominal cavity, for cases with advanced disease, is conducive to maintaining the laparotomic approach in the majority of cases, since complete cytoreduction is the most important prognostic factor [9]. Nonetheless, MIS may represent a valid option in selected cases, which would diminish surgical complications [10]. Furthermore, the shorter recovery time after MIS allows an early onset of adjuvant chemotherapy [11].

The different surgical indications in ovarian cancer where MIS may be applied includes staging surgery for early stage disease, interval debulking surgery for advanced disease after neoadjuvant chemotherapy, and primary debulking surgery for very selected cases.

The role of MIS for staging surgery (SS) in early-stage ovarian cancer has been subject to a large number of studies. Some of them have shown similar effectiveness as laparotomic procedures, considering the number of resected lymph nodes or risk of upstaging due to tumoral rupture [12,13]. MIS offers a faster recovery, shorter hospital stay, and reduced blood loss. Nonetheless, the majority of studies have limited evidence and might also be influenced by confounding factors [14]. Most studies are founded on retrospective evidence.

Interval debulking surgery (IDS) is the elected treatment when the extension of the disease does not allow a complete surgical removal, so neoadjuvant chemotherapy is administered in order to diminish tumoral burden [15]. MIS has also been postulated for IDS, since the extension of disease is less due to adjuvant treatment, allowing a complete cytoreduction [16]. With this indication, MIS has shown similar prognosis compared to laparotomy, with a lower rate of complications [17]. MIS might be laparoscopic or robotic surgery [18]. Retrospective evidence has shown no difference in 3-year survival among patients that underwent laparotomic compared with MIS IDS [19]. Nevertheless, again, the quality of the studies and its retrospective nature may have influenced these results.

The role of MIS for primary debulking surgery in advanced ovarian cancer is practically limited to evaluate tumor resectability rather than to actually serve as a surgical approach for the surgery [20]. There is scarce evidence regarding its applicability for advanced stages, and it has shown lower rates of lymph node dissection and disease resection compared with laparotomy [21]. There is not adequate evidence that supports the use of MIS for advanced stage disease as a primary treatment [22].

In addition, MIS is related to possible complications inherent to the surgery, such as tumor spillage and port-site metastasis, which also need to be considered when planning ovarian cancer surgery [23,24,25].

Since there is still scarce evidence about the role of the surgical route of approach for the treatment of ovarian cancer, our aim was to evaluate the advantages of MIS in the management of ovarian cancer among young patients.

## 2. Material and Methods

We conducted a multicenter retrospective study with the participation of 55 Spanish hospitals. Patients with diagnoses of invasive ovarian cancer from 2010 to 2019 were collected. Our study intended to analyze characteristics that might be differential in younger patients, so age was an excluding factor. All patients were aged from 18 to 45 years old and were diagnosed with invasive ovarian tumors of any histology with FIGO stage I–IV. Patients outside that age range or with a diagnosis of benign or borderline tumors were excluded. This study was approved by the Hospital Clínico San Carlos Ethics Committee (intern code 20/775-E) and, in order to participate, all collaborating hospitals had to obtain the approval of their own ethics committee.

In order to confirm malignancy, histological analysis was compulsory, so the totality of patients underwent surgery. Surgical planning varied depending on the initial suspicion for malignancy and the extension of disease. Suspicion for malignancy was assessed according to the IOTA criteria, based on ultrasound imaging and tumoral markers. Patients with highly suspicious tumors underwent one surgery with intraoperative frozen section to confirm malignancy and proceeded with the cytoreductive or staging surgery if possible. In advanced tumors, if complete cytoreduction was not possible, surgery was performed with a diagnostic and explorative purpose, and patients were referred to neoadjuvant chemotherapy after confirmation of malignancy.

MIS included laparoscopy or robotic surgery. Selection of the approach was based on surgical intention, suspicion of malignancy, extension of disease, tumoral size, and surgical team experience. After initial surgery, patients could be referred to primary chemotherapy, adjuvant chemotherapy, or observation, based on national guidelines. If neoadjuvant chemotherapy was administered, surgery would be postulated as an interval procedure after three or four cycles, if a complete cytoreduction was possible.

PFS was considered as the time, in months, from the initial diagnosis of ovarian cancer to the date of the recurrence diagnosis. OS was defined as the time, in months, from the first diagnosis until the decease date.

### Statistical Analysis

Absolute frequencies and proportions were used to present categorical variables and mean (standard deviation) was used for continuous variables. Comparisons between groups were performed using the Student’s t-test for comparisons between groups of continuous variables and the chi-squared test or Fisher’s exact test for categorical variables as appropriate. Survival analysis was calculated using the Kaplan–Meier method. The log-rank test was used to assess survival differences between groups by univariate analysis. Multivariate cox proportional hazards regression modeling was used to identify the prognostic clinical/pathological features independently associated with OS and progression-free survival (PFS). All the tests were two-sided, and the alpha error was set at 5%. The analyses were performed using STATA 15.1 (StataCorp LLC, College Station, TX, USA).

## 3. Results

A total of 1144 patients were collected. Among them, 867 (75.8%) underwent laparotomic surgery and 277 (24.2%) were treated by MIS. Histology was epithelial ovarian cancer (EOC) in 992 (86.7%) patients and non-epithelial ovarian cancer (non-EOC) in 152 (13.3%) patients. Early-stage tumors (FIGO stage I and II) were present in 630 (55%) patients and advanced-stage tumors (FIGO stage III and IV) in the remaining 514 (45%) patients. The mean age of the patients was 36.5 (SD 6.4) years old in the MIS group and 38.4 (SD 6.5) years old in the laparotomy group. Suspicion for malignancy was low in 155 (13.5%) patients, intermediate in 244 (21.3%) patients, and high in 675 (59%) patients.

Data such as age, tumor size, FIGO stage, body mass index (BMI), histology, preoperative ASA, and suspicion for malignancy were analyzed and compared between both surgical approaches (Table 1). Statistically significant differences were found in all of them but BMI, ASA, and preoperative ECOG. A detailed FIGO stage is reported in Table 2.

Considering surgical procedure, laparotomy was the elected surgical approach in 345 cytoreductive surgeries, 287 staging surgeries, 142 interval surgeries, and 93 fertility-sparing surgeries. On the other hand, MIS was chosen in 45 cytoreductive surgeries, 155 staging surgeries, 8 interval surgeries, and 69 fertility-sparing surgeries (Table 3). The differences found were statistically significant (*p* < 0.0001)

Complications such as blood loss, length of surgery, and hospital stay were higher in the laparotomy group, and were statistically significant. No significant differences were found in the tumoral rupture rate between groups (Table 4).

A multivariate analysis was conducted to analyze OS based on age, FIGO stage, ASA, histology, tumoral rupture, and surgical approach. Data are shown in Table 5.

**Figure 1:**
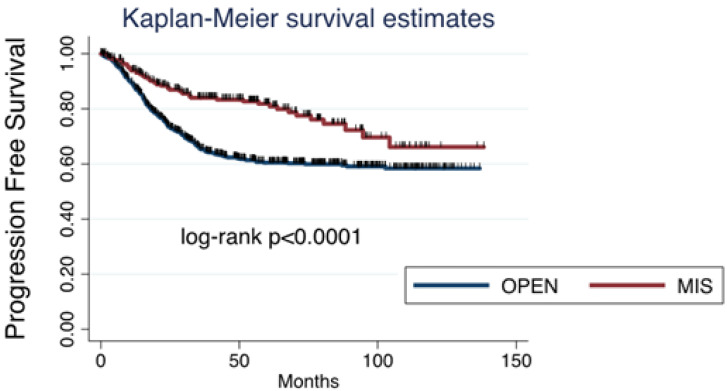
Progression-free survival for ovarian cancer: open surgery versus minimally invasive surgery.

**Figure 2:**
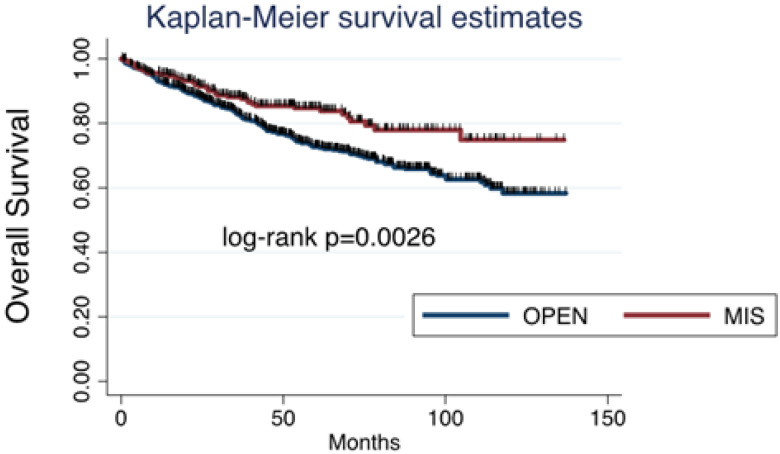
Overall survival for ovarian cancer: open surgery versus minimally invasive surgery.

Among all cases collected, 353 (30.85%) patients had relapse of disease: 302 (26.4%) after open surgery and 51 (4.45%) after MIS. Considering survival, death was reported in 199 (17.4%) patients: 169 (14.8%) in the open surgery group and 30 (2.6%) in the MIS group. Both progression-free survival (PFS) and overall survival (OS) were significantly worse after open surgery than after MIS (Figure 1 and Figure 2).

**Figure 3:**
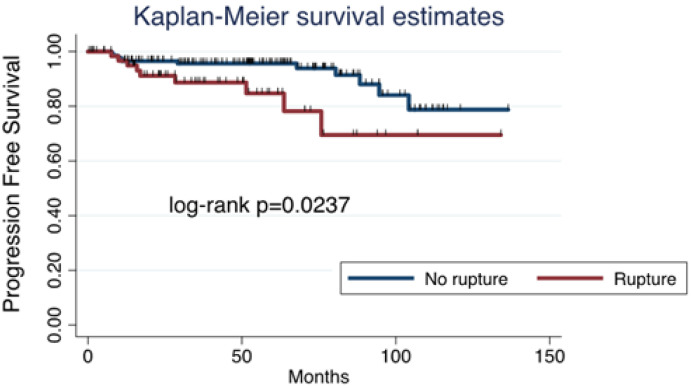
Progression-free survival for minimally invasive surgery: tumoral rupture versus no rupture.

In the MIS group, PFS was influenced by tumor spillage, as it was higher in those tumors that were removed without rupture (Figure 3).

**Figure 4:**
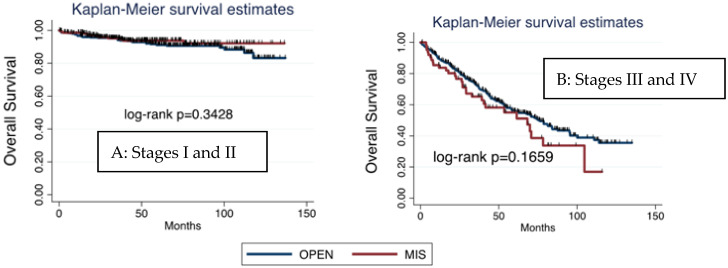
Kaplan–Meier overall survival for FIGO stages I and II ((**A**), left side) versus FIGO stages III and IV ((**B**), right side): open surgery versus minimally invasive surgery. FIGO—Fédération Internationale de Gynécologie et d’Obstétrique.

Considering survival based on FIGO stage and surgical approach, OS was higher for early-stage tumors (FIGO stages I and II), and, within them, the prognosis was better in patients undergoing MIS. On the other hand, OS in advanced stages was lower than in early stage and, in contrast with early stages, was even worse after MIS than after laparotomy. Nonetheless, no significant differences were found (Figure 4).

## 4. Discussion

Our findings prove that MIS does not worsen the prognosis of young patients diagnosed with ovarian cancer when compared with laparotomic surgery. Moreover, we found even lower rates of recurrences and deaths in the MIS group; nonetheless, interpretation of data should be very careful, since the groups were not balanced, so those conclusions are not applicable to the majority of patients. First, most of the patients had undergone laparotomic approach, so both groups presented a clear asymmetry. Second, MIS was mostly used in early-stage tumors, so patients with a greater extension of disease and, therefore, worse prognosis, were more frequently treated with laparotomy. Our findings are consistent with the evidence published so far, which states that the role of MIS for ovarian cancer is still limited, in the majority of cases, to early-stage tumors and staging surgery.

Surgical approach for ovarian cancer surgery has been changing in the past years, with the application of MIS in selected cases [26]. MIS in staging surgery for early-stage tumors has shown its safety when performed by a trained surgeon [27]. In our series, 77.3% of the patients that underwent MIS surgery were early-stage tumors, and 55.95% of the MIS were for staging purposes. After its application for early-stage tumors, effort has also been focused on the role of MIS for IDS and cytoreductive surgery. IDS can be performed by MIS, as stated in the National Comprehensive Cancer Network (NCCN) guidelines, although larger analyses are necessary to assure a good selection of patients [28,29]. In our study, only 5.3% of the IDS were performed by MIS, compared with 94.7% that were laparotomic. Finally, there still lacks evidence to prove the safety of MIS for cytoreduction in advanced stages or recurrences [30,31]. In accordance with this, MIS was elected in only 45 of our 390 cytoreductive procedures, which represents 11.5%.

Indication of MIS for ovarian cancer is highly influenced by factors such as age, tumoral size, FIGO stage, and histology [32]. Laparotomy is more frequent in elder patients, as prognosis improves at a younger age [33]. Our study collected only patients younger than 45 years old, so the rate of MIS might be higher than in other series. Tumoral size is related to the risk of tumoral rupture during MIS, as shown in the LOChneSS study [23]. Tumors that underwent MIS surgery were significantly smaller than those treated with laparotomy, in line with the results of other studies [34]. As for histology and FIGO stage, MIS has proven its efficacy and safety in early-stage tumors [35]. On the other hand, its safety for advanced stages has not been proven, since an optimal removal is the most important prognostic factor [36]. In our series, MIS was mainly chosen for early-stage tumors; only 22.7% of the MIS performed were for advanced-stage tumors.

A multivariate analysis was conducted considering those variables, finding statistically significant differences among them regarding overall survival (OS). OS was higher in patients of a younger age, early-stage tumor, with an ASA < 2, or epithelial tumors. On the other hand, non-significant differences in OS were found concerning tumoral rupture and the surgical approach (Table 4).

Recent studies have been postulated to address the deficiencies of previous studies. The LOChneSS study has postulated the effectiveness of a predictive model for ovarian rupture in MIS [23]. LANCE and KGOG are two ongoing randomized studies carried out to compare open vs. MIS, whose results are expected to discern the actual safety of MIS for IDS [37,38].

## 5. Conclusions

MIS has been predominantly used for staging purposes in early-stage tumors, proving its oncological safety when no spread of disease is presumed. Significant differences concerning OS have been found comparing MIS with open surgery, although both groups are not homogeneous, with a striking increase in advanced stages in the open surgery group, whose bias is probably responsible for the results obtained, so they should be interpreted with caution. Nonetheless, MIS has proven to have a role in the treatment of ovarian cancer, without compromising its oncological safety, but with an extremely cautious selection of cases. Current studies have failed to prove the widespread safety of the use of MIS for ovarian cancer, beyond its use in selected cases [39]. Currently, laparotomy remains the standard of care for ovarian cancer surgery, as provided in ESMO and ESGO guidelines [40]. More studies and the addition of prospective evidence are both necessary in order to identify those patients who will benefit from the advantages of MIS without compromising their oncological safety. Hopefully, the ongoing studies will add high quality evidence to our clinical practice to homogenize the use of minimally invasive surgery for selected ovarian tumors.

## 6. Limitations

Our study is retrospective and multicenter, so there might be a variability of criteria depending on the hospital at the time of choosing the route of approach. Moreover, the groups are not balanced, since laparotomy was the preferred surgical approach due to the stage of the disease, with the consequent worse prognosis inherent to the advanced stage in those cases. These factors need to be considered at the time of evaluating our results.

## Figures and Tables

**Table 1:** Comparison of variables depending on surgical approach. SD—standard deviation; MIS—minimally invasive surgery; ASA—American Society of Anesthesiologist; PS ECOG—Eastern Cooperative Oncology Group Performance Status; BMI—body mass index; mm—millimeters; n—number of cases.

	MIS (n = 277)	Laparotomy (n = 867)	*p*-Value
**Age, mean (SD), years**	36.5 (6.4)	38.4 (6.5)	<0.0001
**Tumoral size, mean (SD), mm**	87.2 (56.5)	104.1 (57.7)	<0.0001
**FIGO stage** **Early (I and II)** **Advanced (III and IV)**	214 (77.3%) 63 (22.7%)	416 (48.0%) 451 (52.05%)	<0.0001
**Histology:** **Epithelial** **Non-epithelial**	223 (80.5%) 54 (19.5%)	769 (88.7%) 98 (11.3%)	<0.0001
**BMI**	24.5 (5.3)	25.0 (5.3)	0.2116
**ASA physical status classification system > 2**	16 (5.9%)	77 (9.3%)	0.075
**PS ECOG** **0** **1** **2** **3** **4**	239 (87.8%) 28 (10.3%) 5 (1.8%) 0 (0%) 0 (0%)	675 (85.9%) 95 (12.1%) 8 (1.0%) 3 (0.4%) 5 (0.6%)	0.443
**Malignancy suspicion (based on ultrasound and tumoral markers)** **Low** **Intermediate** **High**	63 (23.5%) 95 (34.5%) 102 (38.1%)	92 (10.9%) 149 (17.6%) 573 (67.7%)	<0.0001

**Table 2:** Detailed description of FIGO stage between groups. n—number of cases.

	Total (n = 1144)	MIS (n = 277)	Laparotomy (n = 867)
IA	276 (24.12%)	109 (39.35%)	167 (19.26%)
IB	29 (2.53%)	11 (3.97%)	18 (2%)
IC1	151 (13.2%)	51 (18.45%)	100 (11.53%)
IC2	60 (5.25%)	15 (5.4%)	45 (5.2%)
IC3	30 (2.62%)	10 (3.6%)	20 (2.3%)
IIA	32 (2.8%)	9 (3.25%)	23 (2.65%)
IIB	52 (4.55%)	9 (3.25%)	43 (5%)
IIIA1	34 (3%)	9 (3.25%)	25 (2.9%)
IIIA2	20 (1.75%)	7 (2.52%)	13 (1.5%)
IIIB	55 (5%)	7 (2.52%)	48 (5.56%)
IIIC	293 (25.6%)	22 (8%)	271 (31.25%)
IVA	46 (4%)	4 (1.44%)	42 (4.85%)
IVB	66 (5.76%)	14 (5%)	52 (6%)

**Table 3:** Description of surgical approach based on surgical intention.

	MIS	Laparotomy
**Cytoreductive surgery**	45 (16.25%)	345 (39.8%)
**Staging surgery**	155 (55.95%)	287 (33.1%)
**Interval debulking surgery**	8 (2.9%)	142 (16.4%)
**Fertility-sparing surgery**	69 (24.9%)	93 (10.7%)

**Table 4:** Complications related to surgery. n—number of cases; SD—standard deviation; mL—milliliters; min—minutes.

	MIS (n = 277)	Laparotomy (n = 867)	*p*-Value
**Blood loss, mean (SD), mL**	239.6 (312.0)	448.7 (415.2)	**<0.0001**
**Surgical time, mean (SD), min**	203.4 (103.3)	246.3 (111.4)	**<0.0001**
**Hospital stay, mean (SD), days**	4.1 (7.7)	8.1 (9.1)	**<0.0001**
**Tumor rupture**	71 (25,6%)	248 (28.6%)	**0.332**
**Adjuvant chemotherapy**	146 (52.7%)	707 (81.54%)	**<0.0001**
**Pregnancies**	24 (8.66%)	23 (2.65%)	**<0.0001**

**Table 5:** Multivariate analysis considering overall survival.

	Hazard Ratio	*p*	95% Conf. Interval
**Age**	1.03	**0.014**	1.006–1.057
**Advanced FIGO stage (III &IV)**	7.19	**<0.001**	5.097–10.155
**ASA**	1.73	**0.002**	1.213–2.457
**Non-epithelial**	0.42	**0.012**	0.216–0.831
**Tumoral rupture**	0.89	0.441	0.568–1.200
**Open/MIS**	1.14	0.474	0.800–1.615

## Data Availability

The original contributions presented in this study are included in the article. Further inquiries can be directed to the corresponding author(s).

## Abbreviations

ASA	American Society of Anesthesiology
BMI	body mass index
ECOG	Eastern Cooperative Oncology Group Performance Status
EOC	epithelial ovarian cancer
FIGO	Fédération Internationale de Gynécologie et d’Obstétrique
IDS	interval debulking surgery
MIS	minimally invasive surgery
Non	EOC: non-epithelial ovarian cancer
OS	overall survival
PFS	progression-free survival
SD	standard deviation
SS	staging surgery
